# Prevalence and risk factors for faecal carriage of Extended Spectrum β-lactamase producing Enterobacteriaceae among food handlers in lower basic schools in West Coast Region of The Gambia

**DOI:** 10.1371/journal.pone.0200894

**Published:** 2018-08-13

**Authors:** Bakary Sanneh, Abou Kebbeh, Haruna S. Jallow, Yaya Camara, Lusubilo Witson Mwamakamba, Ida Fatou Ceesay, Ebrima Barrow, Fatou O. Sowe, Sana M. Sambou, Ignatius Baldeh, Alpha Jallow, Matheu Alvarez Jorge Raul, Antoine Andremont

**Affiliations:** 1 National Public Health Laboratories, Ministry of Health and Social Welfare, Kotu Layout, Kotu, The Gambia; 2 Epidemiology and disease Control Department, Ministry of Health and Social Welfare, Kotu Layout, Kotu, The Gambia; 3 World Health Organization, Inter-country Support Team, Ouagadougou, Burkina Faso; 4 Medical Microbiology Laboratory, Edward Francis Small Teaching Hospital, Banjul, The Gambia; 5 Department of Health Promotion and Education, Ministry of Health and Social Welfare, Kotu Layout, Kotu, The Gambia; 6 World Health Organization, CountryOffice, The Gambia; 7 Food Safety and Zoonoses Department, World Health Organization, Geneva, Switzerland; 8 University Paris-Diderot Medical School, Paris, France; Natural Environment Research Council, UNITED KINGDOM

## Abstract

**Background:**

The isolation of Extended spectrum βlactamase (ESBLs) producing Enterobacteriaceae among food handlers and their implication as sources of food borne outbreaks are a public health concern. This study seeks to investigate the prevalence of faecal carriage of these bacteria among food handlers in the West Coast Region of The Gambia.

**Method:**

This study enrolled 600 participants from 60 Lower Basic Schools in West Coast Region of the country. Stool samples collected from the participants were presumptively screened for the ESBLs producing Enterobacteriaceae, using Drigalski agar, supplemented with 2mg/L cefotaxime. The bacterial colonies that grew on each Drigalski agar were tested for ESBL production by the double disk synergy test as recommended by Clinical and Laboratory Standard Institute (CLSI-2015). The confirmatory analysis for ESBL was determined as the zone of inhibition of cefotaxime and/or ceftazidime to ≥5mm from that of cefotaxime /clavulanicacid and/or ceftazidime/clavulanic acid. The presumptive screening of isolates for AmpC phenotypes was done by testing the organism against cefoxitin. The prevalence of the ESBL carriage was presented in percentages. The association of risk factors to the faecal carriage of ESBLs producing Enterobacteriaceae was performed by Pearson Chi-squared and Fishers Exact at (p ≤ 0.05).

**Result:**

The prevalence of faecal carriage ESBL producing Enterobacteriaceae among food handlers was 5.0% (28/565). We found50% (14/28) and3.57% (1/28) ESBL producing bacteria were presumptive AmpC and carbapenemase resistance phenotype. Themost abundant ESBL producing Enterobacteriaceae were *Klebsiella spp* 32.1% (9/28) and *Escherichia spp* 28.6% (8/28). The use of antibiotics in the last 3 months was found to be significantly associated (P = 0.012) with the faecal carriage of ESBLs producing Enterobacteriaceae.

**Conclusion:**

The prevalence of faecal carriage of ESBLs producing Enterobacteriaceae among food handlers in the Gambia is low. The history to use of the antibiotics in the last three months was found to be significantly associated with this prevalence. Therefore, the institution of a robust antimicrobial surveillance and treatment of patients with such infections are necessary to curb the spread of these multidrug resistant bacteria in the country. Rational prescription and usage of the antibiotics especially cephalosporin should be advocated both in public and private health facilities.

## Background

The treatment of infectious agents with cephalosporin has been followed by the emergence and dissemination of Extended-spectrum β-lactamase (ESBLs) producing bacterial pathogens that confer resistance to third generation cephalosporin [1, 2]. These gram-negative bacteria producing ESBLs have been identified from a range of Enterobacteriaceae in different communities in the world [2]. ESBLs have spread throughout the globe and *Klebsiella pneumoniae* and *Escherichia coli* strains have been reported as the most common producers of these antibiotic resistant enzymes and causing treatment challenges in nosocomial and community-acquired infections[3]. These infections are responsible for increased morbidity and mortality as the management and care call for expensive drugs and prolonged hospitalization[4]. Studies have demonstrated the isolation of ESBL producing Enterobacteriaceae among of healthy people in communities, who were confirmed with long term carriage potential of these bacteria[5, 6, 7]. Moreover, some researchers have shown that ESBL-producing Enterobacteriaceae such as *E*. *coli* lineages have so far been identical in both isolates from animal products and human samples, thus suggesting their probable transmission across livestock and human [7].

The first outbreak involving ESBL-producing Enterobacteriaceae strains was documented in Germany in 1983[8]. Food handlers and animal foods have been implicated in food borne outbreak caused by ESBL producing Enterobacteriaceae strains in countries such as Spain [9]. Furthermore, a hospital outbreak report provided evidence that contaminated food could be a transmissible route for *K*. *pneumoniae* producing ESBL[10]. In Japan, a faecal ESBL producer carriage study among healthy food handlers found that up to 10 carriers of the ESBL producing bacteria carried these resistant gram negative bacteria up to two years [7]. Similarly in Kenya, 3% of faecal ESBL producer carriage among food handlers was documented [11]. Studies in Vietnam and Algeria revealed that animal food products represent the main reservoir of ESBL/pAmpC-producing *E*.*coli* for human infection[12,13]. Poor food handling procedure could have contributed to the contamination of such food products such as sandwiches which serve as transmission vector in those countries.

A polygenetic study have established close relationships between ESBL producing isolates animal based food, seafood, vegetables and human[1,14]. Furthermore, food handlers, hospital staff, patients, animal based food in a hospital setting were found have similar overlapping relationship between ESBL producing Enterobacteriaceae isolates from human and meat [15]. Therefore, food handlers and others are at risk for contracting ESBL producing Enterobacteriaceae through exposure to contaminated environments such as health-care facilities, farms, livestock, pet, food, ESBL producing health carriers and treatment with third-generation cephalosporin antibiotic [16]. Hence food handlers infected with ESBL producing Enterobacteriaceae pose a great threat for the transmission of this AMR bacterium through the food chain in the globe.

In 2011, a food borne studied in The Gambia and Senegal established significant similar detection of virulence genes in salmonella isolates from animal food products and human [17]. Besides, in rural country site, four food handlers of 128 were implicated with carriage of non-typhoid Salmonella enteric strains which were of animal origin [18]. These studies showcased that there exist potential of risk of transmission of antimicrobial multidrug resistance (AMR) food borne pathogens through the food chain in the country. Other clinical studies showed that Enterobacteriaceae spp such *Klebsiella spp*, *Burkholderia cepacia* were the common pathogen causing serious bacterial infection among admitted children at the Peadiatric Department of Edward Francis Small Teaching Hospital (EFSTH) [19,20]. Ninety-five percent of the children admitted were treated with the antibiotics and majority of these clinical isolates were multidrug especially to the ampicillin which is a monobactem among the first line of antibiotic for the treatment of such infections [19, 20, 21]. Similarly, a retrospective review of causes of urinary tract infections among patients seen at Medical Research Council, The Gambia Unit’s laboratory record in 2013, *E*. *coli* and *Klebsiella spp* accounted for 50% and 10.6% among the most common isolated pathogens [22]. Consequently, information from the antimicrobial multidrug resistance sentinel site at EFSTH has shown increased in the isolation of the gram-negative bacteria are resistant to ceftriaxone. These national studies have implicated few of mother to child possible infection routed have been confirmed but other source of transmission such nosocomial and community [19]. Thus, there is limited data for the prevalence of ESBL producing Enterobacteriaceae faecal carriage in The Gambian communities. This study is relevant in establishing the prevalence and risk factors of the Enterobacteriaceae producing ESBL faecal carriage among food handlers in schools, who could serve as potential reservoir for the transmission of these pathogens.

## Methods and materials

### Ethics

The study was approved by The Gambia Government and Medical Research Council Joint ethical committee with a number SCC1416. Written informed consent was obtained from all the enrolled food handlers by their signatures or fingerprints.

### Study populationandsite

Across-sectional study was conducted in West Coast Region of The Gambia from July to September 2015, which comprised of two educational regions; West Coast Region 1 and West Coast Region 2. This region was purposely selected because it’s densely populated due to rural-urban drift for socio-economic and academic reasons. The region also housed most of the industries, social amenities and majority of the public and private health facilities [23]. The Lower Basic Schools (LBS) in this region were stratified into two educational regions, then districts. We randomly selected the schools as per the Ministry of Basic and Secondary Education’s (MoBSE) approach of grouping schools as reported in our study [23]. A total of 60 LBS, 30 from each region were selected randomly for the study to meet the 95% confidence interval of the school population. The sample size was calculated using the formula (n = Z2P (1-P)/d2) as done by Daniel 1999 employing the 65% prevalence of the faecal carriage of ESBL producing Enterobacteriaceae in a community study done in Thailand to yield the sample size [24]. The social mobilization team comprising of staff from Directorate of Health Promotion and Education, Food Safety Authority and Ministry of Basic and Secondary Education were visited with all food handlers registered in the selected schools. Nine food handlers were randomly selected from the list by the social mobilization team and consented to participate in the study at each school. Schools with fewer food handlers were added from the neighboring school food handlers. A faecal sample was collected from each consented food handler.

One stool sample was collected from each food handler in a well labeled transparent and leak proof screw cap plastic container of about 30ml. The samples were immediately recorded by the field staff in the chain of custody form and sample stored at cold chain temperature (2 to 8°C) until delivery at the laboratory within 6 hours. The consented food handlers were interviewed by the field staff with standardized questionnaires looking at their demographic information and other risk factors for the potential carriage of ESBL producing Enterobacteriaceae.

### Laboratory investigation

Time and temperature at which the samples were collected were recorded and signed by the laboratory technician receiving the samples on the chain of custody form. The stool Samples’ processing time and date were recorded in per batch on the sample processing laboratory log book.

### Screening for the presumptive ESBLs

Approximately 1g of stool specimen were initially inoculated into the enrichment media (peptone water) which was incubated for 24hr at 37°C and then 100μl was sub-cultured in a selective Chromogenic agar (Drigalski agar & 2mg of cefotaxime) [25]. The presumptive ESBLs colonies were then further purified in the nutrient agar and then the species were confirmed.

### Phenotypic confirmatory test for ESBLs

The presumptive ESBLs were confirmed for EBSL phenotype using double disc diffusion test and interpreted by following the Clinical Laboratory Standard Institute (CLSI) guidelines [26]. A Pure culture of identified ESBLs was prepared to 0.5 McFarland turbidity standard in 0.85% saline and inoculated by spreading using sterile swabs on Muller Hinton Agar medium.

The following antibiotics were analyzed; cefotaxime (30μg), cefotaxime/clavulanic acid (30 μg/10μg), ceftazidime (30 μg), ceftazidime/clavulanic acid (30 μg/10 μg), were used as recommended by CLSI-2015 to establish the status of the ESBL phenotypes [26]. The confirmatory analysis for ESBL was determined as the zone of inhibition of cefotaxime and or ceftazidime to ≥5mm from that of cefotaxime/clavulanic acid and or ceftazidime/clavulanic acid. The presumptive screening of isolates for AmpC phenotypes was done by testing the organism against cefoxitin. The plates were then incubated aerobically at 37°C for 18 to 24 hours. *Klebsiella pneumoniae* ATCC 700603 (positive control) and *Escherichia coli* strain ATCC 25922 (negative control) were used for quality control [CLSI-2015].

### Antimicrobial susceptibility testing for the ESBL producing isolates

The ESBLs producing Enterobacteriaceae strains were tested for susceptibility to ceftriaxone, cefuroxime, ciprofloxacin, tetracycline, ampicillin, imipenem and cotrimoxazole using the disc diffusion method and applied the CLSI breakpoint for the interpretation of the degree of susceptibility [26].

Statistical analysis: the data was entered into the Epi-Info database and Microsoft Excel version 2010 software which was screened and cross-checked before the analysis. The categorical data were analyzed using the SPSS version 16 software. Pearson Chi-Square and fisher’s exact test by crosstab method was used to determine the association of risk and socio-demographic factors to the faecal carriage of ESBLs Enterobacteriaceae. The statistical significant value was set at P<0.05.

## Results

### Demographic characteristic of the food handlers

Out of the 600 food handlers enrolled, 565 participants had a complete dataset and were analyzed. A total of 561 out of 565(99.3%) of food handlers were females with a mean age of 37years. Most of the study participants stayed less than 5km from the nearest health facility. The majority of food handlers have been selling and preparing food for not more than 4 years and are been certified. Only (17%) of food handlers have been trained on food safety procedure as shown in Table 1.

**Table 1:** Demographic characteristics of the study food handlers in West Coast Region.

Characteristics	Number of respondents	%	P value
Female gender	561	99.3	0.452
Age (mean)yrs	37		0.112
Staying in homes <5km from health facility	551	98.5	0.698
Raring domestic animals.	351	62.1	0.569
Length of handling/selling food for ≤4yrs	409	72.38	0.24
Trained for food handling	101	17.9	0.00
Certified for handling food	504	89.4	0.103
Know the principle of food safety	515	91.2	0.002

The study found that 5.0% (28/565) of food handlers were faecal carriers of Enterobacteriaceae producing ESBL. Of these 28 ESBL producing isolates, 85.7% (24/28) were found in West Coast Region1, whilst 14.3% (4/28) in West Coast Region 2 as shown in Table 2.

**Table 2:** Distribution of ESBL producing Enterobacteriaceae carriage among food handlers in West Region (Region1 and Region 2).

Number tested and confirmed cases of ESBL per Region				
	Number Tested		Number confirmed	
	N	%	N	%
Region 1	266	47.1	24	85.7
Region 2	299	52.9	4	14.3
Total	565	100	28	100

The study found 28 bacteria which produced ESBL of which there were 14 species producing ESBL amongst the food handlers in west coast region of The Gambia. Majority of the species 85.7% (12/14) were belonged to the Enterobacteriaceae. The predominant were *Klebsiella pneumonia* 32.1% (9/28), *E*.*coli* 28.6% (8/28). This study also found that 50% (14/28) of these ESBL producing isolates were presumptiveAmpC producers whilst 14% (1/28) had carbapenemase production potential as shown in Table 3.

**Table 3:** Distribution of the species of ESBL producing Enterobacteriaceae among food handlers in West Coast Region.

Bacterial Isolates	ESBL producing isolates		ESBL producing ampC isolates		ESBL producing Carbapenemase isolates	
	N	%	N	%	N	%
*E*. *coli*	8	28.6	4	14.3	1	3.6
*Klebsiella pneumonia*	9	32.1	4	14.3	0	0
*Enterobacter cloacae*	2	7.1	2	7.1	0	0
*Serratia fonticola*	1	3.6	1	3.6	0	0
*Serratia liquefacieus*	1	3.6	0	0	0	0
*Citrobacter freundii*	2	7.1	2	7.1	0	0
*Cronobacter spp*	1	3.6	0	0	0	0
*Proteus mirabilis*	1	3.6	0	0	0	0
*Vibrio fluvalis*	1	3.6	0	0	0	0
*Pantoea spp*	1	3.6	1	3.6	0	0
*Cedecea davisae*	1	3.6	0	0	0	0
Total	28	100	14	50	1	3.6

All the ESBL producing isolates were found completely resistant to cefotaxime; ceftriaxone and cefuroxime and ceftazidime. Moreover, 96.4% (27/28) of these isolates were resistant to, ampicillin. Resistance to other antibiotics such as ciprofloxacin, tetracycline, cotrimoxazole and nitrofurantoin were found with the following resistance 62.9% (18/28), 89.9% (25/28), 78.6%(22/28), and 67.9% (19/28) respectivelyto these strains. Only 3.6% (1/28) of these ESBL producing isolates were resistant to imipenem as shown in Table 4.

**Table 4:** ESBL producing Enterobacteriaceae resistant pattern to antibiotics among food handlers.

Antibiotics	Resistant		Intermediate		Sensitive	
	Number	Percentage (%)	Number	Percentage (%)	Number	Percentage (%)
Cefotaxime	28	100	0	0	0	0
Ceftriaxone	28	100	0	0	0	0
Ceftazidime	28	100	0	0	0	0
Ceforuxime	28	100	0	0	0	0
Cefoxitin	14	50	0	0	14	50
Imipenem	1	3.6	6	21.4	21	75
Nitrofurantoin	19	67.9	9	32.1	0	0
Ciprofloxacin	18	62.9	5	17.9	5	17.9
Ampicillin	27	96.4	0	0	1	3.6
Tetracycline	25	89.9	1	3.6	2	7.1
Cotrimoxazole	22	78.6	2	7.1	4	14.3

All the *Klebsiellapneumoniae* ESBL producing strains were found completely resistant to the following cephalosporin: cefotaxime, ceftriaxone, ceftazidime and ceforuxime. Besides, these isolates were also found with 100% resistant to other antibiotics such as ampicillin and cotrimoxazole. Our study found that these Klebsiella pneumonia ESBLs producing isolates were also resistant to ciprofloxacin, nitrofurantoin, and tetracycline with following rates 77.8% (7/9), 66.7% (6/9) and 88.9% (8/9) respectively. Only 33.3% (3/9) and 11.1% (1/9) were resistant to cefoxitin and imipenem respectively as in Table 5.

**Table 5:** Antibiotic resistant pattern of *Klebsiella pneumonia* producing ESBLs among food handlers.

Antibiotics	Susceptibility		Intermediate		Resistant	
	N	%	N	%	N	%
Cefotaxime	0	0	0	0	9	100
Ceftriaxone	0	0	0	0	9	100
Ceftazidime	0	0	0	0	9	100
Ceforuxime	0	0	0	0	9	100
Cefoxitin	6	66.7	0	0	3	33.3
Imipenem	8	88.9	1	11.1	0	0
Nitrofurantoin	1	11.1	2	22.2	6	66.7
Ciprofloxacin	1	11.1	1	11.1	7	77.8
Ampicillin	0	0	0	0	9	100
Tetracycline	0	0	1	11.1	8	88.9
Cotrimoxazole	0	0	0	0	9	100

The study found that 100% (8/8) of the *E*.*coli* producing strains were resistant to ceftriaxone, cefotaxime, ceftazidime, cefuroxime, ampicillin and tetracycline as shown in Table 6. These strains were also highly resistant to the ciprofloxacin, cotrimoxazole, and nitrofurantoin with the following rates: 75% (6/8), 87.5% (7/8) and 75% (6/8) respectively. Of 50% (4/8) of these ESBL producing strains were also resistant to cefoxitin. Only 12.5% (1/8) of these strains were found to be resistant to imipenem as in Table 6.

**Table 6:** Antibiotic resistant pattern of *E*. *coli* producing ESBLs among food handlers.

Antibiotic	Susceptibility		Intermediate		Resistant	
	N	%	N	%	N	%
Ceftriaxone	0	0	0	0	8	100
Cefotaxime	0	0	0	0	8	100
Ceftazidime	0	0	0	0	8	100
Ceforuxime	0	0	0	0	8	100
Cefoxitin	4	50	0	0	4	50
Imipenem	5	62.5	2	25	1	12.5
Nitrofurantoin	0	0	2	25	6	75
Ciprofloxacin	1	12.5	1	12.5	6	75
Ampicillin	0	0	0	0	8	100
Tetracycline	0	0	0	0	8	100
Cotrimoxazole	0	0	1	12.5	7	87.5

Most of the demographic characteristics have no strong association with the Enterobacteriaceae producing ESBL. Lack of food handling training and knowledge of the principle of food safety had a strong association with the ESBL carriage with P values of 0.001 and 0.002 respectively. The uses of antibiotics in the last 3 months have shown some significant degree of association as a risk factor to the carriage of this Enterobacteriaceae. Other risks factors such as had diarrhea episodes in the last three months (p = 0.042) and compliance to completion of prescribing antibiotics (p = 0.0555) were found to have some evidence of correlation to this Enterobacteriaceae as inTable 7.

**Table 7:** Risk factors for faecal carriage of ESBL producing Enterobacteriaceae among food handlers in West Coast Region.

Risk factors	Number of respondent	%	P value
Normally wash hands with soap under running water	560	99.1	0.385
Have pit latrine toilet facilities	329	58.3	0.325
Admitted in a hospital in the last three months	13	2.3	0.134
Had medical instrumentation while on admission	4	0.71	0.385
Escorted /visited a sick family member to the hospital in the last three months	84	14.9	0.934
Stayed with somebody who been admitted in the hospital.	89	15.8	0.741
Used antibiotics in the last three months	185	32.7	0.012
Bought and used antibiotics from street vendors	17	3	0.83
Used antibiotics without consultation of medical practitioner	13	2.3	0.418
complete taking your prescribed antibiotics	545	96.4	0.055
Had diarrhea in the last three months	68	12.03	0.042
Had urinary tract infection in last three months	13	2.3	0.968

## Discussion

The study found that 99.3% (561/565) of the food handlers were female in their late 30 years. Most of these food handlers were not trained on food safety procedure although certified for food handling through laboratory screening for salmonella, Shigella and other intestinal parasitic infection within the a period of six months. This finding comparable to that foodhandlers’ study conducted in Kiang West of the country where female folk dominated the food preparation and services which reflected the inherent cultural practice in TheGambia[18]. However, a study reported that male folk dominated the food handlers’ industry in Kenya[11].

The prevalence of faecal carriage of ESBL producing Enterobacteriaceae among these food handlers was 5.0% (28/565). This low prevalence of ESBL faecal carriage is similarto that of the studies in Guyana, Senegal, Japan, Kenya in communities [7,11,27,28]. However, very high prevalence of ESBL carriage were the reports in Thailand, Guinea Bissau, and Tanzania [24, 29, 30]. Majority of these ESBL carriers were found in of West Coast Health Region One which represented the bustling districts with all social amenities. Our findings have shown that ESBL carriers were abundant in cosmopolitan districts whilst very few in remote and less developed districts in the country in as other studies [28,31]. These infected food handlers pose a threat for the spread of the multidrug resistant gram-negative bacteria in schools, their families and communities in which they prepare and serve food. If not treated some of these carriers of ESBLs could serve as long term reservoir for the transmission of these pathogen as demonstrated in a cohort study of food handlers in Japan [7].

We found that *Klebsiella pneumoniae* and *E*. *coli* were the most abundant bacterial species producing ESBLs as shown in Table 3. This finding is similar in the retrospective study of aetiologies of urinary tract infection where the most predominant a etiologies were *E coli* and *K*. *pneumoniae* in the Medical Research Unit laboratory records in 2013[22]. Although many studies have documented *K*.*pneumoniae*infected to be associated with nosocomial route of spread, our report have shownpotential community base transmission route through food handlers. Of these Enterobacteriaceae 50% (14/28) were found to produce AmpC as found in other studies. Our study found few of the isolates 5.5%(1/28) were presumptive carbapenemase phenotypes as was also found in a study in Thailand [24]. This finding shows that potential risk exists for the spread of community acquired urinary tract infections and invasive infection.

Our study found that the Enterobacteriaceae producing ESBLs were all resistant to ceftriaxone, cefuroxime, cefotaxime and ceftazidime.In fact, ceftriaxone is the only 3rd generation cephalosporin available in the country for advanced care and management was found totally resistant to these gram negative bacteria. These ESBL producing bacteria were almost all resistant to the first line of antibiotics for the treatment gram negative as recommended in The national treatmentGuideline[21]. Furthermore, we found that 62.9% of the gram negative bacteria were resistant to ciprofloxacin which is the recommended second of treatment.

This antibiotic resistant trend is comparable to the studies conducted at the paediatric department of EFSTH hospital were ampicillin was the most resistant antibiotic[19,20]. Other studies in the country also discovered highly AMR patterns among salmonella and *Helicobacter pylori* isolates [17,32]. A study had found similarities among isolate of faecal carriage of ESBL with molecular similarities of those causing urinary tract infections and producing ESBL which poses food handlers who are ESBL carrier as potential threat for transmission of infections in communities.

This study has found that the faecal carriage of ESBLs in food-handlers was significantly associated with history of antibiotics usage, history of diarrheal episodes in the last 3 months and lack of knowledge in food safety practices as found in other studies [2,4]. High prescription and usage of the antibiotics have been reported in studies conducted at EFSTH [19, 20]. However, ESBL carriages were not associated with the study conducted in Japan [7]. Admission in the hospital, instrumentation whilst in admission and staying with a hospitalized individual was not significantly associated with the carrier of the ESBL as were demonstrated in a study [4, 33]. This study did not find significant association between ESBL carriage with rearing domestic animals as in other studies [24]. It was found that the proximity to the health facility was also not importantly associated with carriage of such ESBLs as was found in the study in Guinea Bissau [29]. Therefore, treatment of patients with the antibiotics such as cephalosporin increases the risk for acquiring ESBL carriage and hence it will be prudent for the country to institutes stewardship for rationale antibiotic prescriptions and usage among health workers and communities.

### Limitation

The limitations of this study were the lack of a molecular confirmatory test to establish genotypes of ESBLs in The Gambia, however, the studies from west Africa found the CTXM-15, and CTXM-1 are the most common [29, 28]. Also, the participants were unable to provide their medical records in order to confirm the prescribed antibiotics in order to show association with the faecal carriage of ESBLs. Most of the study participants were female which tend to show bias to the gender though some studies had found ESBL carriage to be more associated with male. The data analysis associated risk factor for ESBL carriage was limited to chi square, fishers’ exact analysis with less than p value less 0.05 as significant relationship. One rare ESBL bacteria with susceptible to a monobactem may require further investigation. The study design was limited because there was no follow-up to treat ESBL carrier and find how they could remain the infections as done in Japan[7].

## Conclusion

The study for the first of its kind has established low prevalence of ESBL faecal carriage of Enterobacteriaceae in the food-handlers in the country who could pose a threat for onward transmission of such infection if not treated. It was also found that most of the antibiotics available for the treatment of these gram-negative bacterial infections were resistant to these ESBL phenotypes thus posing a threat to the management of such infections in The Gambia. Use of antibiotics and lack of knowledge on food safety were major risk factors for the carriage of the ESBL. It will be ideal to strengthen the antimicrobial surveillance system and regular screening of clinical isolates production of ESBLs and its treatment and control strategies. Antibiotic stewardship should be advocated amongst health care workers and the communities should be sensitized prudent use of antibiotic.

## Supporting information

S1 Table An anynanimous data set of the study was subjected as submission supporting documents (S1 Table.ESBL PROJECT AMR DATA SET 2015). (XLSX)
